# Survival of veterans treated with enzalutamide and abiraterone for metastatic castrate resistant prostate cancer based on comorbid diseases

**DOI:** 10.1038/s41391-022-00588-5

**Published:** 2022-09-14

**Authors:** Martin W. Schoen, Kenneth R. Carson, Seth A. Eisen, Charles L. Bennett, Suhong Luo, Melissa A. Reimers, Eric M. Knoche, Alison L. Whitmer, Yan Yan, Bettina F. Drake, Kristen M. Sanfilippo

**Affiliations:** 1 Saint Louis Veterans Affairs Medical Center, Saint Louis, MO USA; 2 Department of Internal Medicine, Saint Louis University School of Medicine, Saint Louis, MO USA; 3 Rush University, Chicago, IL USA; 4 Department of Medicine, Washington University School of Medicine, Saint Louis, MO USA; 5 University of South Carolina College of Pharmacy, Columbia, SC USA; 6 Department of Surgery, Washington University School of Medicine, Saint Louis, MO USA

**Keywords:** Outcomes research, Cancer therapy

## Abstract

**Background:**

Comorbid diseases influence patient outcomes, yet little is known about how comorbidities interact with treatments for metastatic castrate-resistant prostate cancer (mCRPC). No head-to-head trials have compared the efficacy of abiraterone and enzalutamide - oral androgen-receptor targeted agents (ARTAs) for mCRPC. In patients with comorbid disease, outcomes with ARTAs may differ due to disparate mechanisms of action, adverse events, and drug interactions.

**Methods:**

Retrospective observational study of US veterans initiating treatment for mCRPC with abiraterone or enzalutamide between September 2014 and June 2017. Treatment duration and overall survival (OS) was compared based on age and comorbid diseases. The association between ARTA and OS was assessed using Cox proportional hazards and propensity-score matched modeling while adjusting for potential confounders. Sensitivity analyses were performed based on patient age, comorbidities, and subsequent treatments for mCRPC.

**Results:**

Of 5822 veterans treated for mCRPC, 43.0% initially received enzalutamide and 57.0% abiraterone. Veterans initially treated with enzalutamide versus abiraterone were older (mean 75.8 vs. 75.0 years) with higher mean Charlson comorbidity index (4.4 vs. 4.1), and higher rates of cardiovascular disease or diabetes (74.2% vs. 70.6%). In the entire population, veterans initially treated with enzalutamide had longer median OS compared to those initially treated with abiraterone (24.2 vs. 22.1 months, *p* = 0.001). In veterans with cardiovascular disease or diabetes, median treatment duration with enzalutamide was longer (11.4 vs. 8.6 months, *p* < 0.001) with longer median OS compared to abiraterone (23.2 vs. 20.5 months, *p* < 0.001). In a propensity score matched cohort, enzalutamide was associated with decreased mortality compared to abiraterone (HR 0.90, 95% CI 0.84–0.96).

**Conclusions:**

Veterans with cardiovascular disease or diabetes had longer treatment duration and OS with enzalutamide compared to abiraterone. Further study of ARTA selection may benefit men with metastatic castrate resistant prostate cancer and likely hormone sensitive prostate cancer, especially among patients with comorbid diseases.

## Introduction

Metastatic castrate resistant prostate cancer (mCRPC) remains difficult to treat despite availability of multiple therapies. The oral androgen-receptor targeting agents (ARTAs) abiraterone and enzalutamide improve overall survival (OS) in mCRPC [1, 2] and due to more tolerable safety profiles compared to cytotoxic chemotherapy such as docetaxel, ARTAs are frequently administered to patients with comorbid diseases treated within the Veterans Health Administration (VHA) [3]. Although there have been small trials testing drug sequencing, no large clinical trial has compared outcomes between ARTAs [4, 5]. Meta-analyses have not revealed large differences in disease response with these agents [6–8] and they are used interchangeably due to similar results from trials that included patients with few comorbid diseases [9].

Comorbid diseases and age can interact with treatments to affect survival in prostate cancer [10–12]. High rates of short-term mortality and hospitalization have been noted among prostate cancer patients with cardiovascular disease receiving ARTAs [13]. Because the abiraterone and enzalutamide have different mechanisms of action, in patients with complex medical conditions, the drugs may have disparate treatment outcomes and adverse events [14]. Abiraterone acetate inhibits steroid hormone biosynthesis to reduce production of androgens, requires co-administration of prednisone to prevent adrenal insufficiency, and may interact with diabetes [1]. In contrast, enzalutamide blocks hormone signaling directly by inhibiting the androgen receptor and does not require prednisone [2]. These differences may explain the increased rate of hospitalizations in patients treated with abiraterone [15, 16] and increased acute kidney injury, hepatic injury, and atrial fibrillation [17]. Since many men with prostate cancer have cardiovascular disease with inadequately controlled risk factors [18], understanding the interaction between cardiovascular disease and ARTAs may guide treatment decisions.

Large data analyses of real-world treatment can inform clinical practice in situations of clinical equipoise and identify potential differences in survival among patients who are not routinely included in clinical trials. Prior studies showed benefit of enzalutamide over abiraterone using real-world data from the VHA [19] and a French cohort [20]. However, analyses of patients with comorbidities such as cardiovascular disease and diabetes have not been performed and could determine which patients benefit from careful selection of ARTA therapy. Since many veterans with prostate cancer have multiple comorbidities, data from the VHA provides a crucial opportunity to compare treatment duration and survival in high-risk patient populations with cardiovascular disease or diabetes.

## Methods

### Study population

The Veterans Health Administration Informatics and Computing Infrastructure (VINCI) system contains clinical and administrative data from the Corporate Data Warehouse (CDW) from veterans treated across the United States. Using VINCI, we identified patients with an initial prescription for abiraterone or enzalutamide between September 10, 2014 and June 2, 2017. During these years, these medications had the same FDA indications for treatment of mCRPC and were similarly priced with no generic available. On June 2, 2017, abiraterone was reported to improve survival in metastatic hormone sensitive prostate cancer, therefore patients starting treatment after that date were not included [21]. This study was reviewed by the Saint Louis Veterans Affairs Medical Center Institutional Review Board and was performed in accordance with the Declaration of Helsinki.

### Measurements and covariates

We obtained ICD-9/10 codes and laboratory data with the VINCI platform. Pathologic data from the cancer diagnosis was obtained from CDW oncology files, including Gleason score. Pharmacy Benefits Management (PBM) records were accessed to ascertain treatments for all other types of prostate cancer therapy including androgen deprivation therapy (ADT), chemotherapy, and bone-directed therapy of zoledronic acid or denosumab. ADT included treatment with leuprolide, triptorelin, goserelin, histrelin, and degarelix, or ICD codes for orchiectomy. Treatment start date was determined as the first date of the initial prescription for abiraterone or enzalutamide. Patients were considered to have received one treatment for mCRPC if they were prescribed either abiraterone or enzalutamide only and were not prescribed docetaxel, cabazitaxel, mitoxantrone, radium-223, or sipuleucel-T. Patients were considered to have received multiple therapies for mCRPC if they were prescribed two or more of the following: abiraterone, enzalutamide, docetaxel, cabazitaxel, mitoxantrone, or sipuleucel-T.

Baseline demographics collected include race and age at first prescription for abiraterone or enzalutamide. Body-mass index (BMI) was calculated using height and weight within one month of initial treatment. Laboratory data included prostate specific antigen (PSA), albumin, creatinine, and hemoglobin closest to and prior to treatment start, within six months of therapy initiation. Creatinine clearance was calculated using the CKD-Epi formula. The Romano adaption of the Charlson comorbidity index (CCI) [22] and the Quan modification of the Elixhauser index [23] were calculated based on two ICD9/10 codes obtained any time prior to initiation of abiraterone or enzalutamide. Cardiovascular disease was identified by the presence of any ICD codes in the Charlson and Elixhauser indices for myocardial infarction, heart failure, cardiac arrhythmia, valvular disease, complicated hypertension, peripheral vascular disease, and cerebrovascular disease. Diabetes mellitus was identified by ICD codes for diabetes or complicated diabetes. Sites of metastases was determined by presence of ICD9/10 codes in one year prior to treatment initiation [24] and time from first diagnosis was calculated from first pathologic diagnosis of prostate cancer available in CDW-Oncology Raw.

### Outcome measures

Date of death was identified using the VHA Vital Status File and OS was calculated as the time from date of initial prescription of abiraterone or enzalutamide to date of death or April 30, 2020 the last date of follow up. Duration of initial treatment was calculated from the difference in days between the first and last prescription plus the number of days of treatment in the last prescription.

### Statistical analyses

Demographic and clinical characteristics were compared between patients using Chi-square, Student’s t, or Wilcoxan two-sample tests as appropriate. Cox proportional hazards regression modeling was used to assess the association between initial treatment and OS while adjusting for confounding variables. The following variables were included in the full model: age, CCI, black race [25], BMI [26], prior treatment with docetaxel, PSA at time of first treatment, prior bone-directed therapy, bone-directed after starting ARTA as a time-varying variable, anemia (hemoglobin < 10 g/dL), impaired creatinine clearance (CrCL < 30 mL/min), bilirubin ≥2 mg/dL and albumin ≤3 g/dL. Propensity score matching was also performed using age, heart failure, cardiac arrythmia, uncomplicated hypertension, valvular abnormality, myocardial infarction, complicated diabetes, uncomplicated diabetes and renal failure variables to match patients and determine outcome with abiraterone or enzalutamide. Sub-group analyses of patients who had a pathologic record of prostate cancer available were performed to identify patients who primarily received treatment within the VHA.

SAS 9.4 (SAS Inc., Cary NC) and SPSS (IBM, Armonk NY) were used for analyses and to create figures. All *p*-values were 2-tailed with *p* < 0.05 determined as significant.

## Results

From September 10, 2014 to June 2, 2017, 5822 veterans with prostate cancer were initially treated with abiraterone or enzalutamide by VHA medical facilities. The mean age of patients included was 75.4 years, 1351 (23.2%) were African American, and mean CCI was 4.2 (standard deviation 2.7). A comparison of baseline patient characteristics is shown in Table 1. Fewer patients were initially treated with enzalutamide than abiraterone (2504 vs. 3318) and 2804 (48.2%) received only one of the two drugs during the study period. Veterans first treated with enzalutamide were older (75.8 vs. 75.0 years, *p* = 0.002), had higher mean CCI (4.4 vs. 4.1, *p* < 0.001), and had lower median baseline PSA values (27.8 vs. 32.0, ng/mL *p* = 0.03). There were no significant differences in treatment based on race/ethnicity or use of docetaxel prior to treatment with abiraterone or enzalutamide.

**Table 1:** Baseline Characteristics of US Veterans treated with abiraterone and enzalutamide in prostate cancer from 9/10/2014 to 6/2/2017.

	Total (*N* = 5822)		*P*-value
Demographic clinical characteristics	first prescribed abiraterone *n* = 3318	first prescribed enzalutamide *n* = 2504	
**Age (mean years)**	75	75.8	0.002^a^
**Charlson Comorbidity index**	4.1	4.4	< 0.001^a^
**Elixhauser comorbidities**	5.9	6.3	0.002^a^
**Race (%,** ***n*)**			0.91^b^
**White**	75.0 (2487)	74.6 (1867)	
**Black**	23.1 (765)	23.4 (586)	
**Other**	1.8 (60)	1.9 (48)	
**Unknown**	0.2 (6)	0.1 (3)	
**eGFR (%)**			0.003^b^
**>** **=** **30** **mL/min**	90.2	88.3	
**<30** **mL/min**	3.8	3.4	
**Unknown**	6.1	8.3	
**Anemia (%)**			< 0.001^b^
**Hgb** > **=10** **g/dL**	79.3	75	
**Hgb** < **10** **g/dL**	11.2	10.6	
**Unknown**	9.5	14.4	
**Bilirubin (%)**			0.045^b^
**<2**	88.9	86.9	
**>=2**	0.2	0.4	
**Unknown**	11	12.7	
**Albumin (%)**			0.37^b^
**>3** **g/dL**	81	79.6	
**<=3** **g/dL**	6	6.3	
**Unknown**	13	14.2	
**Docetaxel prior (%,** ***n*)**	10.3 (343)	9.0 (224)	0.08^b^
**Docetaxel ever (%)**	28.4	24.4	< 0.001^b^
**Bone directed therapy prior (%,** ***n*)**	23.5 (780)	21.2 (530)	0.03^b^
**Bone directed therapy after (%,** ***n*)**	39.9 (1324)	37.1 (930)	0.03^b^
**Gleason score (n)**	7.8 (1602)	7.9 (1139)	0.23^c^
**PSA closest to start of ARTA (median,** ***n*)**	32.0 (3063)	27.8 (2245)	< 0.001^c^
**Received both abiraterone and enzalutamide (%,** ***n*)**	50.6 (1678)	45.0 (1126)	< 0.001^b^
**Received subsequent treatment for mCRPC (%,** ***n*)**	54.9 (1821)	49.1 (1230)	< 0.001^b^
**Time from diagnosis to start of ARTA (mean months,** ***n*)**	93.8 (2076)	91.0 (1508)	0.29^a^
**Time between start of ARTA and subsequent treatment for mCRPC (mean months)**	13.7	15.3	< 0.001^a^
**Metastatic site 1 year prior (%)**			
**Bone**	44.7	42.3	0.07^b^
**Visceral**	7.5	7.1	0.50^b^
**Lung and Liver**	4.0	4.2	0.59^b^
**Visceral other than Lung and Liver**	3.8	3.9	0.93^b^
**Unspecified**	6.6	6	0.40^b^

^a^T-test.

^b^Chi-square test.

^c^Wilcoxon Two-Sample Test.

ARTA: androgen receptor targeting agent.

### Treatment pattern

**Fig. 1:**
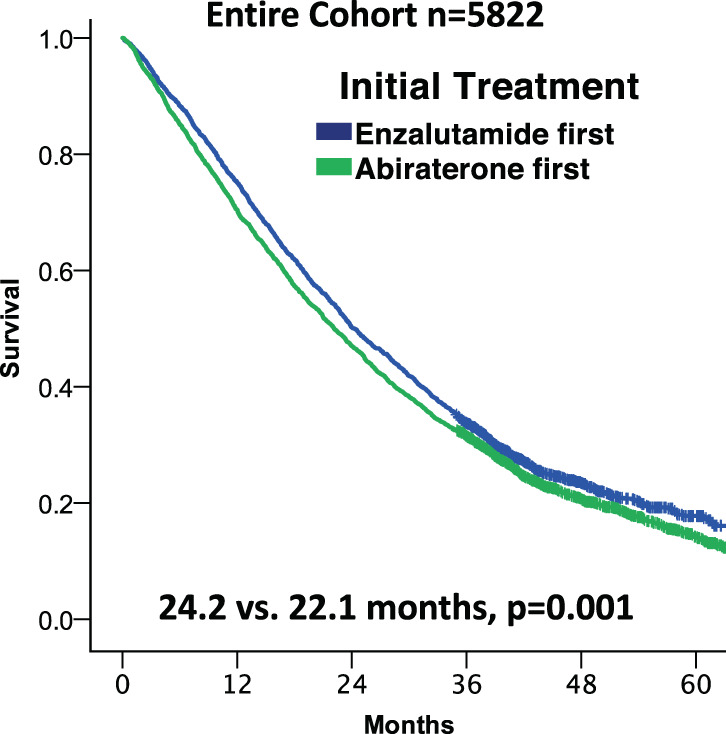
Kaplan-Meier plot of overall survival of all patients based on initial treatment with either enzalutamide or abiraterone. Median months of survival reported. *P*-values calculated via Log-rank.

Table 2 shows treatment duration and OS among all patients and key mCRPC patient subgroups. During a median follow up period of 23.2 months, duration of treatment for veterans initially treated with enzalutamide was 2.6 months longer compared to patients treated initially with abiraterone (11.7 vs. 9.1, *p* < 0.001), and veterans treated with enzalutamide had 2.1 months longer median OS (24.2 vs. 22.1 months, *p* = 0.001) (Fig. 1). Significantly fewer veterans treated with enzalutamide received subsequent treatment for mCRPC (49.1% vs. 54.9%, *p* < 0.001), and time from the start of initial ARTA to subsequent treatment was longer with enzalutamide (15.3 vs. 14.7 months, *p* < 0.001).

**Table 2:**
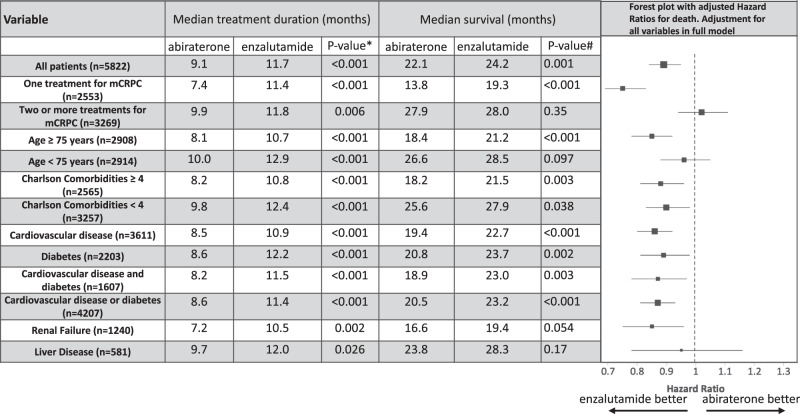
Treatment duration and survival of U.S. Veterans treated for mCRPC between 9/10/2014 to 6/3/2017.

### Age and comorbidities

**Fig. 2:**
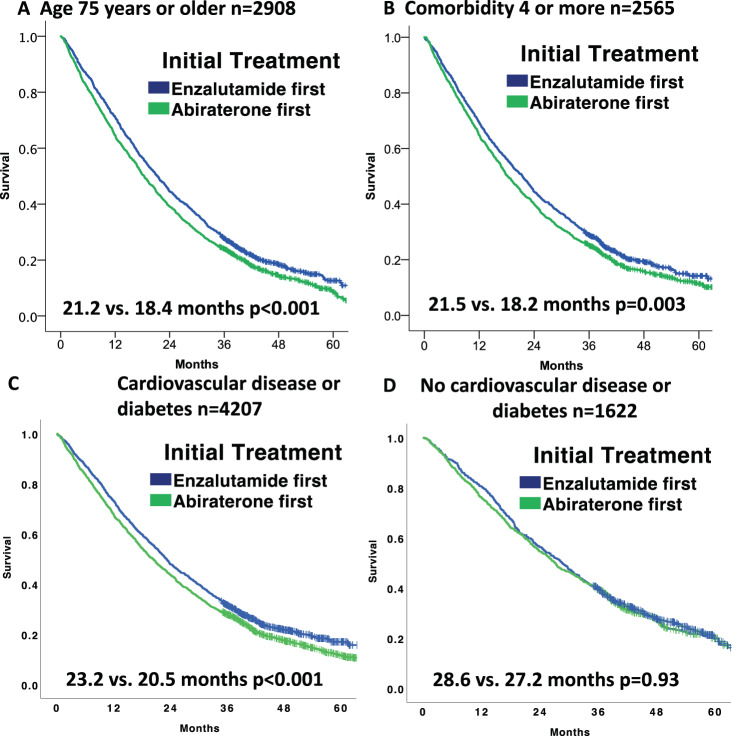
Kaplan-Meier plots of overall survival in subgroups of patients treated initially with enzalutamide or abiraterone for mCRPC with median months of survival reported. *P*-values calculated via Log-rank. **A** 2908 patients aged 75 years or older when first treated. **B** 2565 patients with a Charlson comorbidity index of four or more. **C** 4207 patients with cardiovascular disease or diabetes. **D** 1622 patients without cardiovascular disease or diabetes.

Because age and comorbid diseases effect treatment duration and survival in prostate cancer, we conducted analyses in these important sub-groups. Among 2908 patients age 75 years or older at start of ARTA therapy, median treatment duration with enzalutamide was 2.6 months longer compared to abiraterone, with 2.8 months longer median survival (21.2 vs. 18.4 months, *p* < 0.001, Fig. 2A). Among the 2565 patients with a CCI of 4 or higher, median treatment duration with enzalutamide was 2.6 months longer compared to abiraterone, with 2.3 month longer median survival (21.5 vs. 18.2 months, *p* = 0.003, Fig. 2B). Among 3611 patients who only had cardiovascular disease (61.9% of the entire cohort), median treatment duration with enzalutamide was 2.4 months longer compared to abiraterone with 3.3 months longer median OS (22.7 vs. 19.4 months, *p* = 0.006). Additionally, among 2203 veterans with diabetes (37.8%), median treatment duration with enzalutamide was 3.6 months longer compared to abiraterone with 2.9 months longer median OS (23.7 vs. 20.8 months, *p* = 0.002). In 4207 patients who had either cardiovascular disease or diabetes, median treatment duration with enzalutamide was 2.8 months longer compared to abiraterone with 2.7 months longer median OS (23.2 vs. 20.5 months, *p* < 0.001, Fig. 2C). In 1622 patients without cardiovascular disease or diabetes, there was no difference in survival (28.6 vs. 27.2 months, *p* = 0.93, Fig. 2D).

In an exploratory analyses, veterans who received only one treatment in addition to ADT for mCRPC were compared to veterans who received multiple subsequent treatments for mCRPC. Among 2550 veterans (43.8%) who received only an ARTA for mCRPC and no further treatments, median treatment duration with enzalutamide was 4.0 months longer compared to abiraterone (11.4 vs. 7.4 months, *p* < 0.001), with 5.5 months longer median OS (19.3 vs. 13.8 months, *p* < 0.001, Supplemental Fig. 1A). Veterans who received only one mCRPC treatment were older (78.2 vs. 73.1 years, *p* < 0.001) with higher mean CCI (4.6 vs. 3.9, *p* < 0.001) than veterans who received two or more mCRPC therapies. Among 3272 veterans (56.2%) who received two or more treatments for mCRPC, median treatment duration with enzalutamide versus abiraterone was longer (11.8 vs. 9.9 months, *p* = 0.006); however there was no significant difference in median survival (28.0 vs 27.8 months, *p* = 0.34, Supplemental Fig. 1B).

### Multivariable models

To account for baseline differences between veterans, we created both a Cox proportional hazards and propensity score matched model. In the multivariable Cox model, enzalutamide was associated with 11% decreased mortality versus abiraterone (adjusted Hazard Ratio (aHR) 0.89, 95% CI 0.84–0.95). In 4207 (72%) patients with cardiovascular disease and diabetes, enzalutamide was associated with a 13% decreased mortality (aHR 0.87, 95% CI 0.81–0.93, Table 3). Using age and cardiac comorbidities, a propensity score matched model with mCRPC was created (Supplementary Table 1). In 5006 matched Veterans, enzalutamide was associated with 10% decreased mortality versus abiraterone (aHR 0.90, 95% CI 0.84–0.96) and an increased treatment duration of 1.6 months (15.0 vs. 13.6, *p* < 0.001). In the matched cohort, enzalutamide was associated with 2.1 months longer survival than abiraterone (24.2 months vs. 22.1, *p* < 0.001).

**Table 3:** Association between patient and treatment characteristics and mortality in U.S. Veterans treated for mCRPC.

Variable	All Veterans *n* = 5822		Veterans with cardiovascular disease or diabetes *n* = 4207	
	aHR^a^	95% CI	aHR^a^	95% CI
Enzalutamide vs. Abiraterone	0.89	0.84–0.95	0.87	0.81–0.93
Age (per year increase)	1.02	1.01–1.02	1.01	1.01–1.02
Charlson Comorbidity Index, per point	1.05	1.04–1.06	1.04	1.03–1.06
Race: Non-black	Ref		Ref	
Black	0.75	0.70–0.80	0.76	0.70–0.83
PSA Category: 0–4	Ref		Ref	
4–10	1.18	1.03–1.36	1.18	1.01–1.39
10–20	1.46	1.28–1.67	1.37	1.18–1.60
20–50	1.63	1.44–1.84	1.52	1.32–1.76
50–100	2.12	1.86–2.41	2.05	1.76–2.38
100–200	2.38	2.07–2.74	2.23	1.90–2.63
200 +	2.80	2.45–3.20	2.84	2.42–3.32
unknown	2.08	1.75–2.47	1.85	1.50–2.27
Prior docetaxel	1.36	1.23–1.50	1.28	1.14–1.43
Prior bone-directed therapy	0.91	0.84–0.99	0.78	0.68–0.91
Bone-directed therapy after	1.17	1.09–1.25		
BMI Category: < 18.5	1.63	1.34–1.99	1.49	1.19–1.87
18.5 ≤ BMI < 25	Ref	0.72–0.84	Ref	0.73–0.87
25 ≤ BMI < 30	0.78	0.62–0.73	0.79	0.60–0.72
BMI ≥ 30	0.70	0.74–0.93	0.66	0.69–0.91
BMI unknown	0.83		0.79	
Hemoglobin: Hgb ≥ 10.0 g/dL	Ref		Ref	
Hgb < 10 g/dL	1.83	1.66–2.02	1.73	1.55–1.94
unknown	0.87	0.76–1.00	0.87	0.74–1.03
eGFR: ≥ 30 ml/min	Ref		Ref	
< 30	1.08	0.93–1.25	1.03	0.88–1.21
unknown	1.01	0.82–1.24	1.07	0.84–1.37
Bilirubin: <2 mg/dL	Ref		Ref	
≥ 2 mg/dL	1.94	1.12–3.34	1.43	0.71–2.86
unknown	1.03	0.89–1.18	1.08	0.92–1.26
Albumin: > 3.0 g/dL	Ref		Ref	
≤ 3.0 g/dL	1.85	1.61–2.13	1.88	1.64–2.15
unknown	1.10	0.97–1.25	1.15	0.99–1.32

^a^*aHR* Adjusted Hazard Ratio from Cox proportional hazard model. Variables include: Use of abiraterone vs. enzalutamide, Age, Charlson Comorbidity index Race (black vs. non-black), baseline, *PSA* Prior treatment with docetaxel, treatment with bone directed therapy (zoledronic acid or denosumab), treatment with bone-directed therapy after ARTA (time varying), BMI, baseline hemoglobin (hgb), eGFR, bilirubin, albumin.

### Sensitivity analyses

To account for differences among veterans with mCRPC who were treated within the VHA versus those receiving care at non-VHA facilities, a cohort of veterans who had pathology results in VHA was evaluated. Among the 3584 veterans with pathology results (61.5%), initial treatment with enzalutamide versus abiraterone was associated with 2.7 months longer median treatment duration (12.1 vs. 9.4 months, *p* < 0.001) and 1.7 months longer median OS (24.7 vs. 23.0 months, *p* = 0.025), similar to the overall findings. Additionally, in 2625 patients with who had pathologic results and had cardiovascular disease or diabetes, initial treatment with enzalutamide versus abiraterone was associated with 2.8 months longer median treatment duration (11.9 vs. 9.1 months, *p* < 0.001) and 2.3 months longer median OS (23.6 vs. 21.3 months, *p* = 0.002).

## Discussion

In this large, nation-wide study of mCRPC, US veterans with pre-existing cardiovascular disease or diabetes who received enzalutamide had longer treatment duration and median overall survival compared to those who received abiraterone. This difference highlights an important heterogeneity that can be used by clinicians to select treatments in patients with comorbid diseases. A similar difference was noted in older veterans (≥75 years of age) and veterans with a Charlson comorbidity index of ≥4, in whom treatment with enzalutamide had longer treatment duration and OS compared to abiraterone. In multivariable models and propensity-score matched analyses, initial use of enzalutamide was also associated with a reduced risk of death, particularly in patients with cardiovascular disease or diabetes.

The increased treatment duration and longer OS with enzalutamide may result from improved efficacy or fewer adverse events, especially among veterans with cardiovascular disease and other comorbid diseases [13, 27]. Clinical trials of abiraterone and enzalutamide excluded patients with uncontrolled hypertension and clinically significant heart disease, therefore outcomes in these patients are unknown [1, 2]. In prior studies of mCRPC, treatment with enzalutamide was associated with lower rates of myocardial infarction, stroke, new or worsening hypertension, heart failure, arrhythmia, and diabetes compared to abiraterone [28–30]. Observational studies have linked uncontrolled diabetes with poor response to abiraterone [31]. In our study, we identified an important interaction between veterans with mCRPC and cardiovascular disease or diabetes resulting in decreased survival with abiraterone, highlighting potential toxicity or decreased efficacy. In these veterans, enzalutamide was associated with a median of 2.4 months longer (28%) duration of treatment and a median of 3.3 months longer survival (17%).

Our analyses highlight additional important associations between other patient factors and OS. Black veterans (*n* = 1351, 23%) had improved survival (aHR 0.75) similar to other analyses [32] but were 3 years younger (mean 72.9 vs. 76.1 years, *p* < 0.001). While these differences have been proposed to be from testosterone levels and response to hormone treatment [33], prior studies of chemotherapy have also shown improved survival in Black patients with mCRPC [34] and improved survival and younger age at diagnosis in Black men is observed in lung cancer [35] and hematologic malignancies in the VHA [36, 37]. It is also interesting to note that increased BMI was associated with improved survival, which has been previously been seen in clinical trials in mCRPC [26, 38] and observational data in veterans with non-metastatic CRPC [39], but not in localized disease [40].

It is important for clinicians to consider the first choice of ARTA in a sequence of care. In this study, 52.4% of veterans with mCRPC received subsequent therapy after an ARTA, similar to other studies [41]. In veterans who received only one treatment and never received docetaxel previously, enzalutamide was associated with 5.5 months longer median survival, a 40% increase versus abiraterone. It is possible that veterans who never received subsequent treatment either died or were medically unfit for therapy due to adverse events or high comorbidity burdens. Among veterans with mCRPC who received abiraterone first and then received a second mCRPC treatment, there was no difference in OS compared to veterans who received enzalutamide. In this situation, the veterans who receive a second treatment could be considered to ‘cross-over’ and benefit from salvage treatment for mCRPC. Enzalutamide has activity after abiraterone in randomized trials [4] and observational studies [42]. However, sequential treatment with a second ARTA is not ideal if the patient is eligible for cytotoxic chemotherapy and maximizing the benefit of the first therapy is appropriate [43].

There are scant randomized, comparative data to inform treatment selection for patients with mCRPC. Furthermore, there are no published randomized trials that include patients with significant comorbidities similar to patients with mCRPC treated in the real world. Future trials comparing ARTAs are unlikely to identify differences in survival among patients of older age and/or with comorbid diseases (the subset of veterans associated with the largest treatment benefit), since these patients are unlikely to be included [5]. Therefore, observational large data analyses have the potential to guide treatment where clinical equipoise exists and comparative data is unavailable. Our study builds on prior studies [19, 20] by comparing abiraterone and enzalutamide treatment during a time period when the drugs had the same FDA indications, includes patients who received prior chemotherapy, and provides multiple analyses and models that adjust for a range of patient factors. By focusing on patients who are older and/or who have comorbid illnesses, our analyses give potentially actionable information from real-world data to guide treatment decisions.

## Limitations

The primary limitation of this study is its retrospective observational design and unmeasured confounding. However, we adjusted our analyses for known confounders, performed sensitivity analyses, and included a time period where the two treatments had the same FDA-approved indications, similar rates of use, and no publicly reported studies had identified benefit of either agent among prostate cancer patients with hormone sensitive disease [44]. While there may be unknown biases in treatment selection, in patients without diabetes or cardiovascular disease, or who received two or more treatments, there were no differences in outcomes between the ARTAs, which supports the hypothesis that these medicines can be used interchangeably and have little differences in outcomes in fit patients. In addition, our data is subject to errors associated with studies of administrative datasets where diagnoses and comorbidities were based on ICD-9 or ICD-10 codes. We also performed analysis of veterans with pathologic diagnoses of mCRPC, excluding veterans who received care at non-VA medical centers and therefore a complete record of comorbid diseases may not be available.

## Conclusions

These findings suggest that initial treatment with enzalutamide for mCRPC was associated with 2–3 months longer treatment duration and 10–20% overall longer survival compared to abiraterone. The benefit of enzalutamide was most notable among veterans with cardiovascular disease or diabetes, were 75 years or older, or with high comorbidity burdens. These data may guide treatment in metastatic hormone sensitive prostate cancer, where ARTAs are now commonly used and comorbidities likely have a similar impact on outcomes and adverse events in patients treated outside of clinical trials. Future studies are warranted to assess the effect of age and comorbidities on treatment duration and survival among men with metastatic hormone sensitive prostate cancer.

### Supplementary information

Supplementary Table 1

Supplementary Figure 1

## Data Availability

The dataset supporting the conclusions of this article will be shared upon request and execution of an agreement of use. Members of the scientific community can request a copy of the final dataset by e-mailing Dr. Martin Schoen at martin.schoen@va.gov. The investigator should state their reason for requesting the data and analysis plans. De-identified data will be provided after requestors sign a letter of agreement detailing the mechanisms by which the data will be kept secure and access restricted to their study team. The agreements will also state the recipient will not attempt to identify any individual whose data are included and will not share the data with anyone outside of their research team. Acknowledgement and attribution of scholarly effort is expected for any publication arising from shared data.
