# Correction to: Survival of veterans treated with enzalutamide and abiraterone for metastatic castrate resistant prostate cancer based on comorbid diseases

**DOI:** 10.1038/s41391-023-00680-4

**Published:** 2023-05-30

**Authors:** Martin W. Schoen, Kenneth R. Carson, Seth A. Eisen, Charles L. Bennett, Suhong Luo, Melissa A. Reimers, Eric M. Knoche, Alison L. Whitmer, Yan Yan, Bettina F. Drake, Kristen M. Sanfilippo

**Affiliations:** 1 Saint Louis Veterans Affairs Medical Center, Saint Louis, MO USA; 2 Department of Internal Medicine, Saint Louis University School of Medicine, Saint Louis, MO USA; 3 Rush University, Chicago, IL USA; 4 Department of Medicine, Washington University School of Medicine, Saint Louis, MO USA; 5 University of South Carolina College of Pharmacy, Columbia, SC USA; 6 Department of Surgery, Washington University School of Medicine, Saint Louis, MO USA

**Keywords:** Outcomes research, Cancer therapy

Correction to: *Prostate Cancer and Prostatic Diseases* 10.1038/s41391-022-00588-5, published online 14 September 2022

The article Survival of veterans treated with enzalutamide and abiraterone for metastatic castrate resistant prostate cancer based on comorbid diseases, written by, Martin W. Schoen, Kenneth R. Carson, Seth A. Eisen, Charles L. Bennett, Suhong Luo, Melissa A. Reimers, Eric M. Knoche1, Alison L. Whitmer, Yan Yan, Bettina F. Drake and Kristen M. Sanfilippo was originally published electronically on the publisher’s internet portal on 14 September 2022 without open access. With the author (s)’ decision to opt for Open Choice the copyright of the article changed on 8 May 2023 to © The Authors 2023 and the article is forthwith distributed under a Creative Commons Attribution 4.0 International License, which permits use, sharing, adaptation, distribution and reproduction in any medium or format, as long as you give appropriate credit to the original author(s) and the source, provide a link to the Creative Commons licence, and indicate if changes were made. The images or other third party material in this article are included in the article’s Creative Commons licence, unless indicated otherwise in a credit line to the material. If material is not included in the article’s Creative Commons licence and your intended use is not permitted by statutory regulation or exceeds the permitted use, you will need to obtain permission directly from the copyright holder. To view a copy of this licence, visit http://creativecommons.org/licenses/by/4.0. Open Access This article is licensed under a Creative Commons Attribution 4.0 International License, which permits use, sharing, adaptation, distribution and reproduction in any medium or format, as long as you give appropriate credit to the original author(s) and the source, provide a link to the Creative Commons license, and indicate if changes were made. The images or other third party material in this article are included in the article’s Creative Commons license, unless indicated otherwise in a credit line to the material. If material is not included in the article’s Creative Commons license and your intended use is not permitted by statutory regulation or exceeds the permitted use, you will need to obtain permission directly from the copyright holder. To view a copy of this license, visit http://creativecommons.org/licenses/by/4.0.

