# Supplementary material for: Survival of veterans treated with enzalutamide and abiraterone for metastatic castrate resistant prostate cancer based on comorbid diseases
Source: Prostate Cancer Prostatic Dis. 2022 Sep 14;26(4):743–50. doi: 10.1038/s41391-022-00588-5 (PMC10638085; doi:10.1038/s41391-022-00588-5)
Supplement: Supplementary file 1 — Supplementary Table 1 [file 41391_2022_588_MOESM1_ESM.docx]

| **Table 1: Comparison between first prescribed abiraterone and enzalutamide in prostate cancer propensity score matched cohort from 9/10/2014 to 6/2/2017** | | | | | |  |  |  |
| --- | --- | --- | --- | --- | --- | --- | --- | --- |
|  |  |  |  |  |  |  | | |
|  | **Total (N=5006)** | | | | **P-value** |  | | |
| **Demographic clinical characteristics** | **first prescribed abiraterone n=2503** | | **first prescribed enzalutamide n=2503** |  | |  |  |  |
| **Age (mean years)** | 75.7 | | 75.8 | 0.60† | |  |  |  |
| **Elixhauser comorbidity index** | 6.2 | | 6.3 | 0.35† | |  |  |  |
| **Charlson score (mean)** | 4.2 | | 4.4 | 0.16† | |  |  |  |
| **Race (%)** |  | |  | 0.82* | |  |  |  |
| **White** | 75.5 | | 74.6 |  | |  |  |  |
| **Black** | 22.6 | | 23.4 |  | |  |  |  |
| **Other** | 1.7 | | 1.9 |  | |  |  |  |
| **Unknown** | 0.2 | | 0.1 |  | |  |  |  |
| **EGFR (%)** |  | |  | 0.36* | |  |  |  |
| **>=30** | 88.4 | | 88.3 |  | |  |  |  |
| **<30** | 4 | | 3.4 |  | |  |  |  |
| **Unknown** | 7.6 | | 8.3 |  | |  |  |  |
| **Anemia (%)** |  | |  | 0.0003* | |  |  |  |
| **HgB>=10** | 79 | | 75 |  | |  |  |  |
| **HgB<10** | 10.2 | | 10.6 |  | |  |  |  |
| **Unknown** | 10.8 | | 14.4 |  | |  |  |  |
| **Bilirubin (%)** |  | |  | 0.32* | |  |  |  |
| **<2** | 87.6 | | 86.9 |  | |  |  |  |
| **>=2** | 0.2 | | 0.4 |  | |  |  |  |
| **Unknown** | 12.2 | | 12.7 |  | |  |  |  |
| **Albumin (%)** |  | |  | 0.82* | |  |  |  |
| **>3** | 79.4 | | 79.5 |  | |  |  |  |
| **<=3** | 6 | | 6.3 |  | |  |  |  |
| **Unknown** | 14.6 | | 14.2 |  | |  |  |  |
| **Docetaxel prior (%, n）** | 9.7 (243) | | 9.0 (224) | 0.36* | |  |  |  |
| **Docetaxel ever (%, n)** | 26.4(661) | | 24.3(609) | 0.09* | |  |  |  |
| **Bone agent (%)** | 5.4 | | 7.6 | 0.001* | |  |  |  |
| **Gleason score (mean, n)** | 7.8 (1183) | | 7.9 (1138) | 0.14‡ | |  |  |  |
| **PSA closest to start of ARTA (median, n)** | 27.6 (2249) | | 27.8 (2244) | 0.89‡ | |  |  |  |
| **Received both abiraterone and enzalutamide (%, n)** | 49.3 (1234) | | 45.0 (1125) | 0.0002 | |  |  |  |
| **Received subsequent treatment for mCRPC (%, n)** | 53.8 (1346) | | 49.5 (1239) | 0.003* | |  |  |  |
| **Time between start of ARTA and subsequent treatment for mCRPC (mean months)** | 13.4 | | 15.0 | <0.0001† | |  |  |  |
| *** Chi-square test** | | | | | | | |  |
| **† T-test** | | | | | | | |  |
| ‡ Wilcoxon Two-Sample Test  ARTA: androgen receptor targeting agent | |  |  |  |  |  |  |  |
