# Supplementary figures and images for: Survival of veterans treated with enzalutamide and abiraterone for metastatic castrate resistant prostate cancer based on comorbid diseases
Source: Prostate Cancer Prostatic Dis. 2022 Sep 14;26(4):743–50. doi: 10.1038/s41391-022-00588-5 (PMC10638085; doi:10.1038/s41391-022-00588-5)

**Supplement Figure 1A 1 treatment n=2550**

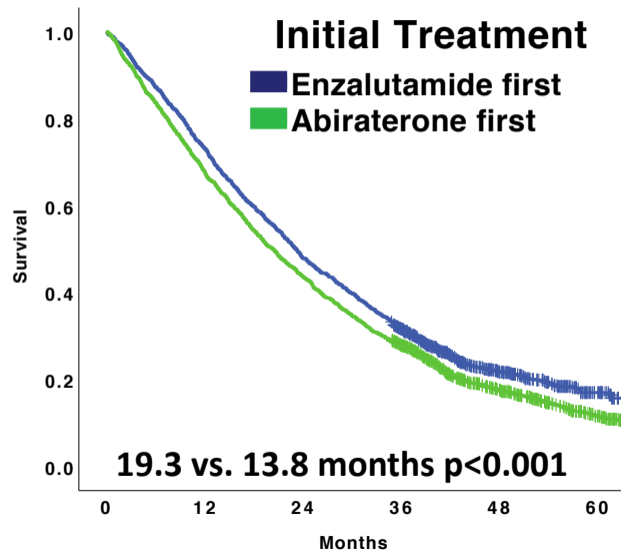

**1B 2+ treatments n=3272**

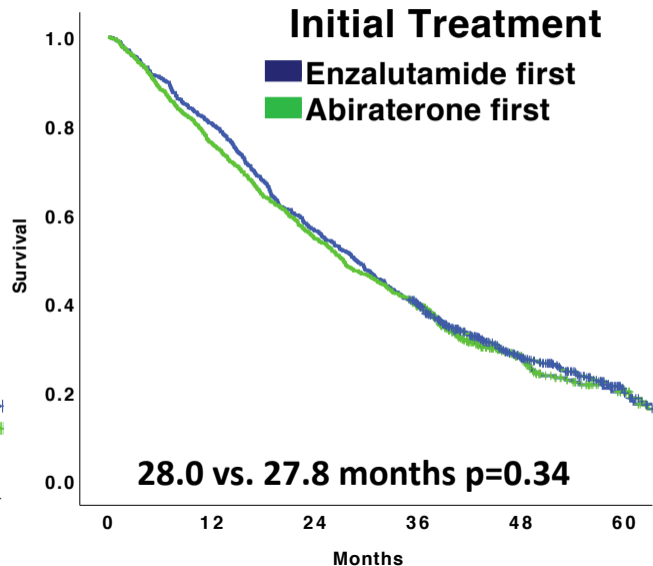

Supplement: Supplementary file 2 — Supplementary Figure 1 [file 41391_2022_588_MOESM2_ESM.pdf]
